# Empirical evidence of disease activity thresholds used to indicate need for major therapeutic change in US veterans with rheumatoid arthritis

**DOI:** 10.1186/s13075-020-02346-1

**Published:** 2020-10-22

**Authors:** Brian C. Sauer, Wei Chen, Yizhe Xu, Jincheng Shen, Neil A. Accortt, David H. Collier, Grant W. Cannon

**Affiliations:** 1 Salt Lake City VA Medical Center and University of Utah, Salt Lake City, UT USA; 2 Veterans Affairs Salt Lake City Health Care System, University of Utah School of Medicine, Salt Lake City, UT USA; 3 Amgen Inc., Thousand Oaks, CA USA

**Keywords:** ACR20, CDAI, DAS28, RAPID3, Major therapeutic change, Rheumatoid arthritis, VARA registry, Youden Index

## Abstract

**Background:**

A previous analysis of the Veterans Affairs Rheumatoid Arthritis (VARA) registry showed that more than half of the patients with rheumatoid arthritis (RA) did not receive a major therapeutic change (MTC) despite moderate or severe disease activity. We aimed to empirically determine disease activity thresholds associated with a decision by rheumatologists and nurse practitioners to institute a MTC in patients with RA and to report the impact of that change on RA disease activity.

**Methods:**

We analyzed data from the VARA registry between January 1, 2006, and September 30, 2017. Eligible patients had a visit with 3 disease activity measures (DAMs) recorded: Disease Activity Score for 28 joints (DAS28), Clinical Disease Activity Index (CDAI), and Routine Assessment of Patient Index Data 3 (RAPID3). The Youden Index was used to identify disease activity thresholds that best discriminated rheumatologist/nurse practitioner decision to initiate MTC. Clinical outcome was 20% improvement in the American College of Rheumatology criteria (ACR20 response). The effect of MTC on ACR20 response was presented as crude descriptive statistics and evaluated using G-computation for marginal and conditional effects with established disease activity level combined with an empirical threshold from Youden analysis.

**Results:**

The study population comprised 1776 patients (12,094 visits: 3077 with MTC, 9017 without MTC). Empirical thresholds (95% bootstrap confidence interval with 1000 replications) for MTC were 4.03 (3.70–4.36) for DAS28, 12.9 (10.4–15.4) for CDAI, and 3.81 (3.32–4.30) for RAPID3. Visits with MTC had increased likelihood of ACR20 response: risk ratios for ACR20 response for visits with MTC vs without MTC ranged 1.2–2.6 across DAMs; risk differences ranged 0.2–14.5%.

**Conclusions:**

MTC was associated with clinical improvement across all DAMs with the greatest change in patients with RA disease activity above the Youden threshold identified in this work.

**Trial registration:**

VARA Registry, https://www.hsrd.research.va.gov/research/abstracts.cfm?Project_ID=2141698764

## Introduction

Both the American College of Rheumatology (ACR) [1] and the European League Against Rheumatism (EULAR) [2] recommend assessment of disease activity as part of rheumatologists’ and nurse practitioners’ treatment decisions for patients with rheumatoid arthritis (RA). This recommendation encourages rheumatologists and nurse practitioners to accurately determine the level of disease activity at each patient visit and adjust therapy to achieve a target of low disease activity or remission. Several disease activity measures (DAMs) have been developed in attempts to quantify disease activity for use in real-world practice as well as in clinical trials to measure treatment benefit [3]. For clinical practice, ACR recommends the use of composite measures, including Disease Activity Score for 28 joints (DAS28), Clinical Disease Activity Index (CDAI), Routine Assessment of Patient Index Data 3 (RAPID3), Patient Activity Scale II (PAS II), and Simplified Disease Activity Index (SDAI), to assess the level of disease activity [4]. Each of these measures provides thresholds for remission, low/minimal, moderate, and high/severe levels of disease activity, but differ in scale and in the components used in the calculation of a composite disease activity score.

The goal of treatment for RA is to achieve sustained remission or low disease activity [1, 2]. In the treat-to-target strategy recommended by ACR and EULAR [1, 2], patients should be followed at regular intervals and their treatment adjusted until the target disease activity is achieved. In our prior work using data from the Veterans Affairs Rheumatoid Arthritis (VARA) registry, we evaluated major therapeutic changes (MTC) among US Veterans with RA across a broad range of disease activity [5]. We found that more than half of the patients in the analysis did not receive a MTC despite moderate or severe disease activity. In a chart review of a subset of these patients, we found that the most common reason for no MTC was that the rheumatologist/nurse practitioner judged the RA to be under good control and thus no change in therapy was indicated. Notably, although rheumatologists and nurse practitioners collected the components needed to calculate DAS28 for each patient, those calculations were often not performed in real time during the clinic visit, resulting in rheumatologists and nurse practitioners to base treatment decisions on their clinical judgment without using DAMs. In subsequent work, we found that patients with a MTC had more frequent clinical improvement as measured by 20% improvement in ACR criteria (ACR20 response) than patients who did not have a MTC, even among patients with long-standing RA who had received multiple prior therapies in this patient population [6]. We also noted that patients with higher disease activity were more likely to receive MTC from the rheumatologist/nurse practitioner [5].

In the current analysis, we further explored the relationship between established DAM thresholds of disease activity and rheumatologist/nurse practitioner decision to initiate a MTC. The two goals of this study were to (1) empirically determine the disease activity thresholds at which the rheumatologists and nurse practitioners were most likely to initiate MTC in the VARA population and (2) to report clinical response observed after a MTC based on such thresholds. For the first objective, the Youden Index [7] was used to determine the DAM threshold that best discriminated the decision to initiate a MTC. For the second objective, we estimated the impact of MTC on ACR20 response across 4 categories of disease activity levels as measured by DAS28, CDAI, and RAPID3: remission/low disease; low-moderate disease based on the lower bound of moderate disease to the Youden-identified threshold; high-moderate disease, which ranged from the Youden-identified threshold to the high bound of moderate disease activity; and high disease activity.

## Methods

### Population, data source, and study design

The study population included US Veterans enrolled in the VARA registry [7–10], a prospective, observational registry involving 11 Veterans Affairs (VA) medical centers. DAM components are recorded during routine visits using templated notes. The DAMs are extracted from medical notes stored in the VA Corporate Data Warehouse (CDW) [11] using validated extraction algorithms or data entered manually into the VARA registry database.

Patient data extracted from the CDW include pharmacy, laboratory, outpatient diagnoses, and electronic medical notes. Patient demographics, disease history, and duration of RA were collected from VARA enrollment data. Serologic samples collected to assess rheumatoid factor (RF) and anti-cyclic citrullinated peptide antibodies (ACPA) were assayed at a central laboratory on enrollment into the VARA registry. An additional chart review was performed to collect any data not identified in the CDW or VARA database.

A historical cohort design was used to compare the clinical response between patient visits with and without MTC. The unit of observation was an eligible patient visit to a rheumatology clinic during the study period (January 1, 2006, to September 30, 2017). Each eligible visit (i.e., rheumatology visits with documented core clinical measures to compute DAS28, CDAI, and RAPID3) with 18 months of enrollment and 2 rheumatology visits with DAS28 during the previous 18 months was classified as having a MTC or no MTC. The study included a baseline measurement period (18 months before the eligible visit) to measure covariates and potential confounders and an exposure period (7 days prior to 30 days after the eligible visit) to assess if a MTC occurred. The 7-day pre-visit exposure period was selected to identify any interventions that may have occurred immediately prior to the visit, (e.g., steroid dose escalation via telephone call or electronic message), and the 30-day post-visit period was designed to capture interventions that started at the visit. An outcome period (2–6 months after the eligible visit) was used to identify patients who achieved an ACR20 response (Supplemental Fig. S1).

### Visit eligibility criteria

Eligible visits were identified for patients meeting the following criteria: enrolled in VARA registry, ≥ 18 years of age, rheumatology visit with all components of DAMs (DAS28, CDAI, RAPID3), documented (referenced as an eligible patient visit), ≥ 18 months of enrollment in VA health care system prior to the eligible visit, and 2 rheumatology visits with documented DAS28 scores during the 18-month baseline period ≥ 60 days apart from each other and ≥ 60 days before the eligible patient visit (to measure disease stability) (Supplemental Fig. S1). The key exclusion criteria included active cancer, organ transplant, diagnosis of other autoimmune disorders (e.g., systemic lupus erythematosus), any surgical procedure within 90 days after the eligible visit, or any hospitalization within 30 days of the eligible visit.

### Youden Index and empirical decision threshold

The Youden Index is a measure of diagnostic accuracy that is used to identify optimal thresholds that discriminate a dichotomous outcome from a continuous scale [7]. The Youden Index has traditionally been used to identify optimal cut points for diagnostic tests. In this analysis, the Youden Index was used to identify the DAM value that maximized the correct classification of MTC where equal weighting was given to sensitivity and specificity.

The Youden Index (*J*) was calculated for each cut point/threshold (*c*), i.e., every value of the DAM.

$$ J(c)=\mathrm{sensitivity}(c)+\mathrm{specificity}(c)-1 $$

The goal of this analysis was to maximize *J* to identify the optimal cut point where *c* represents the set of candidate cut points/thresholds:

$$ {c}^{\mathrm{opt}}=\arg\ {\max}_{c\in C}J(c) $$

### Measurements

#### Exposure: MTC

MTC has been previously defined [5, 6]. Briefly, a visit was associated with a MTC if (1) a new disease-modifying antirheumatic drug (DMARD) was initiated (including switching agents within the same drug class) either as a new agent or after a 90-day gap following the last date of prior therapy, (2) DMARD dose was escalated by ≥ 25%, (3) prednisone was initiated, (4) monthly average prednisone dose increased by 25%, and (5) and/or intra-articular injection of ≥ 2 with corticosteroids.

#### Outcome: clinical improvement measured by ACR20 response criteria

An ACR20 response was defined as improvement of 20% in both tender and swollen joint counts and 20% improvement in 3 of the ACR core disease activity measures (patient assessment of pain, patient global assessment of disease activity, physician global assessment, patient assessment of physical function, and acute-phase reactant laboratory value) [12]. ACR20 response was chosen to measure the treatment effects because it is a validated and common outcome measure in clinical trials, has standardized outcome assessments across the DAMs, and can detect clinical response to treatment in a time frame consistent with routine follow-up care (~ 3 months) [13, 14]. A window of 2–6 months after the index visit to document outcomes was used to account for variability in observed visit intervals and reduce the risk of exposure misclassification due to subsequent treatment modification. If multiple visits with documented core clinical measures were observed during the follow-up period, data from the visit closest to 3 months after the index visit were used.

#### Covariates: potential confounders between MTC and ACR20 response

Covariate adjustment was used to remove confounding between MTC and ACR20 response. Potential confounders included demographic characteristics, duration of RA, level of disease activity, Rheumatic Disease Comorbidity Index (RDCI) [15], disease stability [5], DMARD use at baseline, and MTC within 90 days of the eligible visit.

The standard criteria have been established to classify disease activity into remission, low, moderate, and high disease activity (Supplemental Table 1) [16–18]. For this analysis, we also evaluated disease activity stratification that included a division of moderate disease activity based on Youden thresholds. With this method, categories of disease activity included the following: (1) remission and low disease activity for DAS28 (< 3.2), CDAI (< 10.0), and RAPID3 (< 2.0); (2) low-moderate (lower bound of moderate disease to the Youden-identified threshold) for DAS28 (3.20–4.02), CDAI (10.0–12.9), and RAPID3 (2.00–3.81); (3) high-moderate (greater than Youden-identified threshold to the high bound of moderate disease activity) for DAS28 (4.03–5.10), CDAI (13.0–22.0), and RAPID3 (3.82–4.0); and (4) high disease activity for DAS28 (> 5.1), CDAI (> 22.0), and RAPID3 (> 4.0).

Types of MTC were descriptively analyzed based on category: changes in oral prednisone (initiating medication, restarting medication after a gap, and/or increase in medication dose), intra-articular corticosteroid injections, changes in bDMARD, and changes in csDMARD.

#### Estimating the impact of MTC on ACR20 response

Crude (bivariate) associations between MTC and ACR20 response were represented by risk difference (RD) and risk ratio (RR) with 95% confidence intervals (CIs). Impacts of MTC on ACR20 response were further evaluated using G-computation [19, 20] for the marginal and disease activity level conditional effects. The population average generalized estimating equation (GEE) model with an exchangeable correlation structure [21] was used with the G-computation approach to account for within-patient correlation, as multiple visits per patient were possible. Since the G-computation approach allowed us to predict potential outcomes for the entire population under both treatment conditions (with MTC and without MTC), we first built a model using a complete case analysis (i.e., in visits during the follow-up window with documented core measures), and then applied this model to the full population, including those with missing data, to estimate potential outcomes for every patient. We computed 95% CIs using a bootstrapping method, in which the random sampling (1000 samples) was done with replacement [19].

G-computation models were fit using patient age at visit, sex, race, ACPA status, RF status, disease duration, RDCI score, DAM stability (worsening or not), csDMARDs, bDMARDs, and prednisone dispensed in the month prior to visit and in the previous year, and the baseline MTC (MTC during previous 90 days). An interaction term was used to evaluate how the effect of MTC on ACR20 response was modified by different levels of disease activity. The marginal effect (overall effect) was produced by averaging the differences between the potential outcomes under MTC and the potential outcomes under no MTC, accounting for the fact that treatment effects vary across disease activity levels.

The probability of ACR20 response was shown to be independent of follow-up month when conditioning on MTC and levels of disease activity [6]; we therefore did not adjust for the follow-up interval in our ACR20 response model and used the G-computation models to estimate the population-level effects under the assumption of no loss to follow-up.

Descriptive statistics included the number of observations and percentages for dichotomous and continuous variables, and the number of observations, means, standard deviations (SDs), and 95% CIs [11] for continuous variables. We used several statistical software packages for these analyses, including Microsoft SQL Server, SAS version 9.4, and Enterprise Guide version 7.1. Data preparation and statistical analyses were conducted using Stata 14.

## Results

### Study visits

The study population included 1776 patients with 12,094 eligible visits; among these, 9017 visits were without a MTC and 3077 visits had a MTC. Of the 12,094 eligible visits, 7322 were complete cases (had data to calculate DAS28 during the follow-up window) and 4722 visits had missing data. The median number of visits per patient was 5 (interquartile range 3 to 10). Of the 1776 patients included, 588 (33.1%) patients never experienced a MTC, 1050 (59.1%) patients were in both the MTC and non-MTC groups, and only 138 (7.8%) patients were in the MTC group during the study period. A total of 651 patients did not meet the criteria of inclusion and were excluded from the analysis.

### Patient demographic and clinical characteristics

Patients were younger, had a lower percentage of patients of Caucasian race, and had a shorter duration of RA during visits with MTC compared to patients at visits without MTC. The percentage of males, RF-positive status, ACPA-positive status, and disease stability were similar between visits with or without MTC (Table 1). Patients at visits with MTC had lower percentages of recent (within the past month) and established (within the past year) bDMARD and csDMARD dispensing episodes, but had a higher percentage of baseline use of prednisone compared to patients at visits without MTC. Patients at visits with MTC had a lower percentage of MTC during the 90 days prior to the eligible visit compared to patients at visits without MTC during the previous 90 days.

**Table 1:** Demographic and clinical characteristics

	Study population (1776 patients; 12,094 visits)	
	With MTC (*v* = 3077)	Without MTC (*v* = 9017)
Age, mean years (SD) [95% CI]	63.1 (11.0) [62.7–63.5]	65.6 (10.7) [65.4–65.9]
Male sex, *n* (%) [95% CI]	2784 (90.5) [89.4–91.5]	8190 (90.8) [90.2–91.4]
Caucasian race, *n* (%) [95% CI]	2349 (76.3) [74.8–77.8]	7135 (79.1) [78.3–80.0]
RA duration, mean years (SD) [95% CI]	14.0 (11.6) [13.9–14.7]	15.7 (11.7) [15.5–16.0]
RDCI score mean (SD) [95% CI]	2.3 (1.5) [2.2–2.3]	2.3 (1.5) [2.2–2.3]
RF status, *n* (%) [95% CI]		
Positive	2743 (89.1) [88.0–90.2]	8005 (88.8) [88.1–89.4]
Negative	328 (10.7) [9.6–11.8]	995 (11.0) [10.4–11.7]
Missing	6 (0.2) [0.1–0.4]	17 (0.2) [0.1–0.3]
ACPA status, *n* (%) [95% CI]		
Positive	2572 (83.6) [82.2–84.9]	7491 (83.1) [82.3–83.8]
Negative	497 (16.2) [14.9–17.5]	1501 (16.6) [15.9–17.4]
Missing	8 (0.3) [0.1–0.5]	25 (0.3) [0.2–0.4]
DAM stability, *n* (%) [95% CI]		
Better or no change	2146 (69.7) [68.1–71.4]	6299 (69.9) [68.9–70.8]
Worse	931 (30.3) [28.6–31.9]	2718 (30.1) [29.2–31.1]
Recent dispensing episodes^a^, *n* (%) [95% CI]		
bDMARD	348 (11.3) [10.2–12.5]	1396 (15.5) [14.7–16.2]
csDMARD	646 (21.0) [19.6–22.5]	2710 (30.1) [29.1–31.0]
Prednisone	308 (10.0) [9.0–11.1]	731 (8.1) [7.6–8.7]
Established dispensing episodes^b^, *n* (%) [95% CI]		
bDMARD	1171 (38.1) [36.3–39.8]	3808 (42.2) [41.2–43.3]
csDMARD	2609 (84.8) [83.5–86.0]	8104 (89.9) [89.2–90.5]
Prednisone	1628 (52.9) [51.1–54.7]	3580 (39.7) [38.7–40.7]
Baseline MTC before index visit, *n* (%) [95% CI]		
8–30 days	45 (1.5) [1.1–2.0]	137 (1.5) [1.3–1.8]
8–60 days	161 (5.2) [4.5–6.1]	539 (6.0) [5.5–6.5]
8–90 days	292 (9.5) [8.5–10.6]	1134 (12.6) [11.9–13.3]

*ACPA* anti-cyclic citrullinated peptide antibody, *bDMARD* biologic disease-modifying antirheumatic drug, *csDMARD* conventional synthetic disease-modifying antirheumatic drug, *DAM* disease activity measure, *MTC* major therapeutic change, *SD* standard deviation, *95% CI* 95% confidence interval

^a^Recent dispensing episodes were first dispensed between 8 and 30 days of eligible visit

^b^Established dispensing episodes were dispensed between 8 and 370 days of eligible visit

### Youden Index and empirical thresholds

**Fig. 1:**
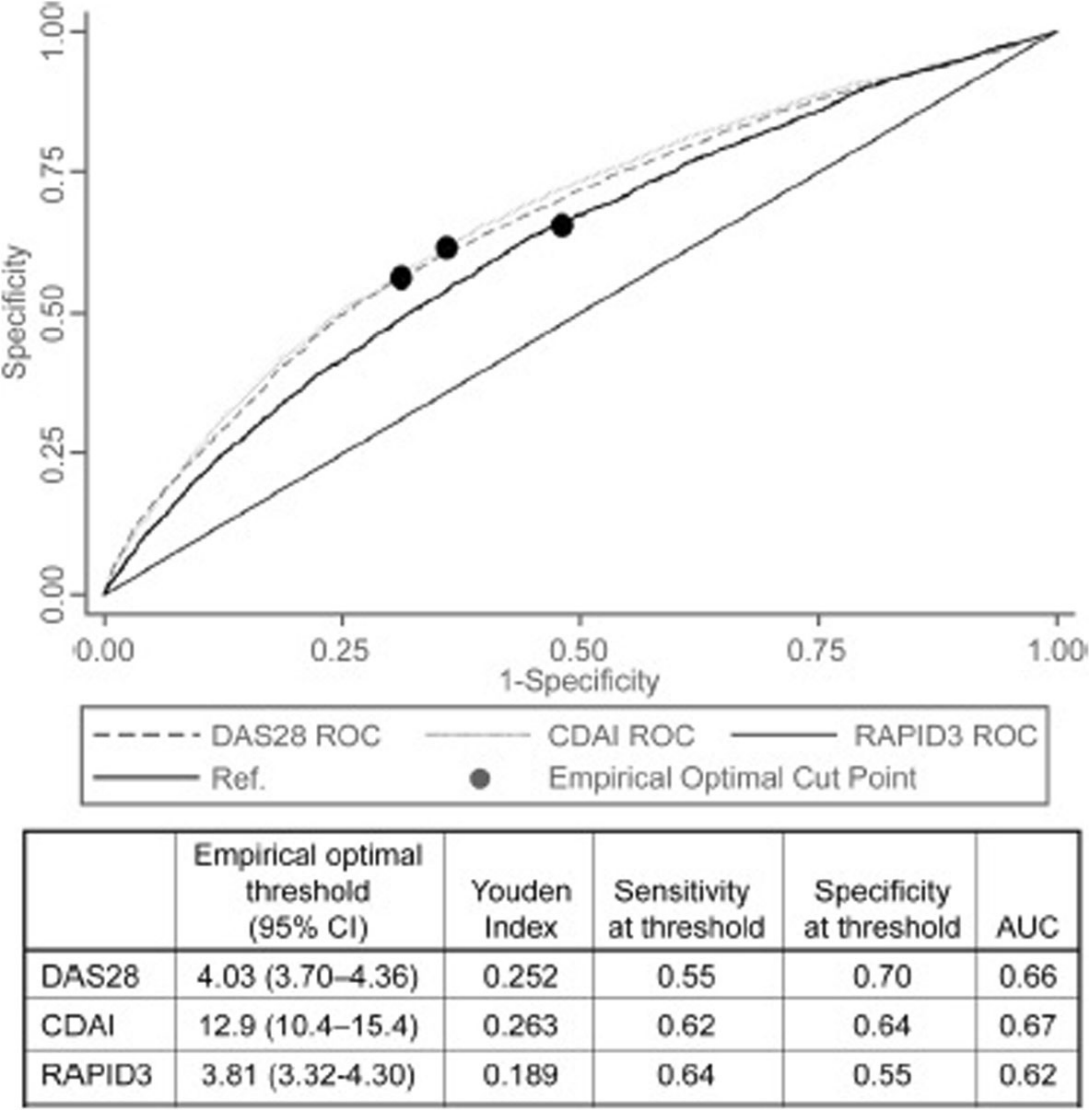
Youden Index and empirical thresholds. ROC curves of disease activity measure thresholds for the likelihood to initiate MTC and empiric thresholds are shown. Empirical optimal thresholds with 95% CI, Youden indices, sensitivity and specificity at each threshold, and the AUC for the ROC curves are reported in the table. 95% CI, 95% confidence interval; AUC, area under the curve; DAS28, Disease Activity Score for 28 joints; CDAI, Clinical Disease Activity Index; MTC, major therapeutic change; ROC, receiver operating characteristic curves; RAPID3, Routine Assessment of Patient Index Data 3

Empirical thresholds for MTC based on the maximum Youden Index and 95% CI were 4.03 (3.70–4.36), 12.9 (10.4–15.4), and 3.81 (3.32–4.30) for DAS28, CDAI, and RAPID3, respectively (Fig. 1, Supplemental Fig. S2).

### Types of MTC

The most common type of MTC across all DAMs and levels of disease activity was changes to csDMARD (Supplemental Table S2). For visits with remission and low, low-moderate, and high-moderate disease activity, the second most common type of MTC was changes to oral prednisone, and for patients with high disease activity, it was changes to bDMARD therapy or to oral prednisone.

### Description of MTC and frequency of ACR20 response

In the crude analysis (complete cases only), visits with remission or low disease activity were generally not associated with MTC, and visits with MTC had low rates of ACR20 responses in these patients (Table 2 and 3. Approximately one fourth to one third of visits with high disease activity with MTC were associated with an ACR20 response (range 22.8–33.5%) regardless of DAM. MTC was generally associated with an increased prevalence of ACR20 response across all strata, but not all strata-specific estimates met statistical significance. When the moderate disease activity category was divided into low-moderate and high-moderate disease based on the Youden threshold, there was a difference in the risk of ACR20 response between the two groups, but the corresponding results on RD did not show a statistically significant difference.

**Table 2:** Crude descriptive analysis of the frequency of MTC and ACR20 response by DAM and disease activity category for visits with ACR20 responses

DAM category	Visits analyzed (7322 complete case visits)*					
	With MTC (*v* = 2113 visits)		Without MTC (*v* = 5209 visits)		RD (95% CI)	RR (95% CI)
	Frequency	ACR20 response frequency (%)	Frequency	ACR20 response frequency (%)		
Overall	2113	400 (18.9)	5209	360 (6.9)	12 (10.21–13.83)	2.7 (2.40–3.13)
DAS28						
Remission	253	4 (1.6)	1217	15 (1.2)	0.3 (− 1.31–2.01)	1.3 (0.43–3.83)
Low	227	9 (4.0)	876	20 (2.3)	1.7 (− 1.04–4.41)	1.7 (0.8–3.76)
Moderate	946	157 (16.6)	2284	183 (8.0)	8.6 (5.96–11.2)	2.1 (1.7–2.53)
High	687	230 (33.5)	832	142 (17.1)	16.4 (12.05–20.77)	2.0 (1.63–2.36)
CDAI						
Remission	90	0	500	0	0	0
Low	397	9 (2.3)	1759	34 (1.9)	0.3 (− 1.27–1.93)	1.2 (0.57–2.43)
Moderate	776	135 (17.4)	1902	154 (8.1)	9.3 (6.36–12.24)	2.1 (1.73–2.67)
High	850	256 (30.1)	1048	172 (16.4)	13.7 (9.89–17.52)	1.8 (1.55–2.18)
RAPID3						
Remission	108	3 (2.8)	444	9 (2.0)	0.8 (− 2.61–4.12)	1.4 (0.38–4.98)
Low	167	16 (9.6)	684	30 (4.4)	5.2 (0.47–9.92)	2.2 (1.22–3.91)
Moderate	526	82 (15.6)	1736	106 (6.1)	9.5 (6.19–12.78)	2.6 (1.95–3.35)
High	1312	299 (22.8)	2345	215 (9.2)	13.6 (11.07–16.17)	2.5 (2.11–2.92)

*95% CI* 95% confidence interval, *ACR20* 20% improvement in American College of Rheumatology criteria, *CDAI* Clinical Disease Activity Index, *DAM* disease activity measure, *DAS28* Disease Activity Score for 28 joints, *MTC* major therapeutic change, *RAPID3* Routine Assessment of Patient Index Data 3, *RD* risk difference, *RR* risk ratio

*The full population included 12,094 visits; the results in this table are based on only complete cases (4772 visits had missing ACR20 responses)

**Table 3:** Crude descriptive analysis of the frequency of MTC and ACR20 response by DAM and disease activity category combined with empirical threshold

DAM category	Visits analyzed (7322 complete case visits)*					
	With MTC (*v* = 2113 visits)		Without MTC (*v* = 5209 visits)		RD (95% CI)	RR (95% CI)
	Frequency	ACR20 response frequency (%)	Frequency	ACR20 response frequency (%)		
Overall	2113	400 (18.9)	5209	360 (6.9)	12 (10.21–13.83)	2.7 (2.40–3.13)
DAS28						
Remission and low (< 3.20)	480	13 (2.7)	2093	35 (1.7)	1.0 (− 0.52–2.59)	1.6 (0.86–3.04)
Low moderate (3.20–4.02)	366	43 (11.8)	1154	57 (4.9)	6.8 (3.28–10.34)	2.4 (1.63–3.47)
High moderate (4.03–5.10)	580	114 (19.7)	1130	126 (11.2)	8.5 (4.79–12.22)	1.8 (1.4–2.22)
High (> 5.10)	687	230 (33.5)	832	142 (17.1)	16.4 (12.05–20.77)	2.0 (1.63–2.36)
CDAI						
Remission and low (< 10.0)	487	9 (1.9)	2259	34 (1.5)	0.3 (− 0.95–1.64)	1.2 (0.59–2.54)
Low moderate (10.0–12.9)	194	18 (9.3)	652	36 (5.5)	3.8 (− 0.69–8.2)	1.7 (0.98–2.89)
High moderate (13.0–22.0)	582	117 (20.1)	1250	118 (9.4)	10.7 (7.03–14.3)	2.1 (1.68–2.7)
High (> 22.0)	850	256 (30.1)	1048	172 (16.4)	13.7 (9.89–17.52)	1.8 (1.55–2.18)
RAPID3						
Remission and low (< 2.00)	275	19 (6.9)	1128	39 (3.5)	3.5 (0.27–6.63)	2.0 (1.17–3.4)
Low moderate (2.00–3.81)	452	73 (16.2)	1555	94 (6.1)	10.1 (6.51–13.7)	2.7 (2.00–3.56)
High moderate (3.82–4.00)	74	9 (12.2)	181	12 (6.6)	5.5 (− 2.75–13.81)	1.8 (0.81–4.17)
High (> 4.00)	1312	299 (22.8)	2345	215 (9.2)	13.6 (11.07–16.17)	2.5 (2.11–2.92)

*95% CI* 95% confidence interval, *ACR20* 20% improvement in American College of Rheumatology criteria, *CDAI* Clinical Disease Activity Index, *DAM* disease activity measure, *DAS28* Disease Activity Score for 28 joints, *MTC* major therapeutic change, *RAPID3* Routine Assessment of Patient Index Data 3, *RD* risk difference, *RR* risk ratio

*The full population included 12,094 visits; the results in this table are based on only complete cases (4772 visits had missing ACR20 responses)

### Marginal and conditional model-based effects of MTC on ACR20 response

In the marginal analysis, the model developed on the complete cases was applied to the full population (12,094 visits). A visit with MTC resulted in a statistically significant greater probability of ACR20 response across all DAMs: RRs for ACR20 response for visits with MTC vs without MTC ranged 1.2–2.6 across DAMs; RDs ranged 0.2–14.5% (Table 4). The stratum-specific effects varied by DAM. MTC was strongly associated with ACR20 response in categories of high disease activity across all DAMs, and again, a marked difference was observed in the percentage of visits with an ACR20 response and RD in the low-moderate disease activity group and high-moderate disease activity group separated by the Youden threshold. In all disease activity categories, MTC consistently showed improvements in ACR20 response in both RD and RR, but not all strata met the statistical significance, which is likely due to the smaller sample size of the study population.

**Table 4:** Adjusted marginal (overall) and conditional (severity category) effects of MTC on ACR20 response for the full population, with the missing ACR20 responses imputed through a causal prediction method

	Visits analyzed (12,094 visits; 1776 patients)*			
	Visits with MTC, % ACR20 response (95% CI)	Visits without MTC, % ACR20 response (95% CI)	RD (95% CI)	RR (95% CI)
DAS28 category				
Overall effect	12.7 (11.2–14.1)	7.3 (6.8–7.0)	5.4 (4.0–6.0)	1.7 (1.5–2.0)
Remission and low (< 3.20)	2.0 (0.9–3.4)	1.2 (0.8–1.7)	0.9 (− 0.4–2.3)	1.7 (0.7–3.4)
Low moderate (3.20–4.02)	10.8 (7.8–14.2)	5.1 (4.0–6.7)	5.7 (2.4–9.3)	2.1 (1.4–3.1)
High moderate (4.03–5.10)	19.7 (16.7–23.2)	12.2 (10.2–14.4)	7.4 (3.8–11.4)	1.6 (1.3–2.0)
High (> 5.10)	34.7 (31.1–38.5)	20.2 (16.9–23.5)	14.5 (10.6–18.7)	1.7 (1.5–2.1)
CDAI category				
Overall effect	12.4 (11.0–13.8)	7.4 (6.9–7.2)	4.9 (3.4–5.5)	1.7 (1.5–1.9)
Remission and low (< 10.0)	1.3 (0.5–2.5)	1.1 (0.7–1.6)	0.2 (− 0.8–1.3)	1.2 (0.4–2.6)
Low moderate (10.0–12.9)	8.2 (4.5–13.0)	5.4 (3.9–7.3)	2.9 (− 1.2–7.7)	1.5 (0.8–2.7)
High moderate (13.0–22.0)	20.3 (17.3–23.8)	10.3 (8.2–12.2)	10.0 (6.3–14.0)	2.0 (1.5–2.5)
High (> 22.0)	31.2 (27.6–34.6)	19.8 (16.9–23.2)	11.4 (7.6–14.9)	1.6 (1.4–1.8)
RAPID3 category				
Overall effect	16.3 (14.5–17.2)	7.0 (6.1–7.6)	9.3 (7.4–10.7)	2.3 (2.0–2.7)
Remission and low (< 2.00)	5.9 (3.5–8.8)	2.9 (2.0–3.9)	3.1 (0.6–5.9)	2.1 (1.2–3.4)
Low moderate (2.00–3.81)	15.2 (12.2–18.4)	5.9 (4.8–7.3)	9.3 (6.1–12.7)	2.6 (1.9–3.4)
High moderate (3.82–4.00)	11.8 (5.2–19.4)	6.5 (3.2–10.1)	5.3 (− 2.7–14.0)	1.8 (0.7–4.3)
High (> 4.00)	22.3 (19.7–24.3)	9.7 (8.4–11.1)	12.5 (9.7–14.9)	2.3 (1.9–2.7)

*95% CI* 95% confidence interval, *ACR20* 20% improvement in American College of Rheumatology criteria, *CDAI* Clinical Disease Activity Index, *DAS28* Disease Activity Score for 28 joints, *MTC* major therapeutic change, *RAPID3* Routine Assessment of Patient Index Data 3, *RD* risk difference, *RR* risk ratio

*Visits with data missing to calculate ACR20 responses imputed using a causal prediction method

## Discussion

In this observational study of US veterans enrolled in the VARA registry, we found that visits with a MTC were associated with an increased likelihood of ACR20 response during the 2–6 months after a MTC was initiated across all DAMs evaluated. However, there was a much greater RD for ACR20 response in patients with disease activity levels above the Youden threshold, as these patients had the greatest potential for response. Disease activity level establishes the indication for MTC and was the strongest predictor of MTC in patients with active disease [5]. The level of disease activity was predicted to act as an effect modifier, as RA patients with stable high disease activity may not respond to treatment, whereas patients with low disease activity or in remission may not receive additional benefit from treatment modification. As we have previously reported [5], clinical characteristics of patients with MTC differed from patients without MTC. Patients with MTC tended to have higher swollen/tender joint counts, greater pain scores, and more severe disease based on patient and physician global assessments of disease. After adjusting for disease activity scores, no clinical or administrative characteristics were predictive of MTC in multivariable regression models in this study population [5].

Disease activity in RA was originally defined as either high (when the rheumatologist/nurse practitioner decided treatment was needed) or low (when the rheumatologist/nurse practitioner decided the patient was in remission, and treatment was no longer needed) [22]. The original response criteria were established using these clinical observations [23]. The DAS28 was designed to measure RA disease activity and assess the treatment response in clinical trials. The development of the instrument was based on statistical analyses of a cohort of patients attending an outpatient clinic at the University Hospital of Nijmegen, and distinguished thresholds for low, moderate, and high levels of disease activity [24]. Current guidelines recommend MTC for patients with moderate and high disease activity [1, 2]. Our prior work and other studies suggest that the thresholds for levels of disease activity are not well aligned with rheumatologist/nurse practitioner current opinions of disease activity in real-world practice [5, 25, 26].

We used the Youden Index to determine how rheumatologists and nurse practitioners discriminated a need for MTC in a population of VARA patients. We identified disease activity scores for each DAM that maximized the discriminant ability for the decision to initiate or not initiate a MTC. The decision to initiate MTC was based on the rheumatologists’ and nurse practitioners’ clinical judgment and assessment of insufficient response. The empiric threshold for each DAM fell within the range of moderate disease. The empiric threshold for DAS28 was 4.03 (range of moderate disease per standard definition ≥ 3.2 to ≤ 5.1), for CDAI was 12.9 (range of moderate disease 10.1 to 22.0), and for RAPID3 was 3.81 (range of moderate disease 2.01 to 4.0) (Supplemental Fig. S2).

Rheumatologists and nurse practitioners consistently judged insufficient response at higher thresholds on the DAM scales compared with the lower threshold in moderate disease, which is the recommended threshold for insufficient response recommended by ACR guidelines. RA is a heterogenous disease, and the trajectory of patients with moderate disease activity varies. In an analysis of data from the Corrona registry, the probability of moving from moderate to low disease between clinic visits was 47%, and from moderate to severe disease, it was 18%, whereas over 35% of patients remained in moderate disease after 6 months [27]. There are currently no reliable methods to predict which trajectory an individual patient’s disease may take, but our results clearly show that patients across the spectrum of disease severity, including those with moderate disease by any DAM definition, can achieve significant clinical benefit from a MTC. Patients with low-moderate disease activity based on the Youden thresholds may therefore represent missed opportunities for clinical improvement with a MTC. In this study, we also observed clinical benefit (ACR20 response) in 7% of the non-MTC group. As in clinical trials when some patients receiving placebo are observed to have a significant clinical improvement, some of the patients in the non-MTC group had an ACR20 response. However, the ACR20 response was much less for the non-MTC group than for the MTC group which demonstrated the clinical benefit of the MTC. While we do not have a complete explanation for the ACR20 response in patients without MTC, we postulate that the reasons for the change in clinical activity would be similar to that seen in placebo control trials which include disease variability and regression to the mean.

The strengths of this work are that all the patients had a rheumatologist-confirmed diagnosis of RA, the study population was nationwide, and clinical and pharmacy data were collected through a uniform medical record and data collection system. Additionally, patients receiving treatment in the VA system benefit from the reliability of prescribing and ready access to medications.

This study cohort comprises US veterans who are predominantly males, with longer disease duration, older age, and more comorbidities than other RA groups, and these results may not be generalizable to the general RA population. These patients with longstanding RA have likely cycled through multiple therapies, yet still realized a clinical benefit with a MTC. A similar analysis in a cohort of newly treated patients with shorter disease duration may show a larger impact with MTC, as rheumatologists and nurse practitioners may be more likely to target remission in patients with shorter duration of RA [28]. This analysis was population-based, whereas rheumatologists and nurse practitioners make decisions for individual patients. For patients with longstanding RA, rheumatologists and nurse practitioners may believe that moderate disease represents the most achievable or best possible outcome based on their understanding of a particular patient; those patients would appear to be undertreated in our analysis.

In summary, these data demonstrate the importance of evaluating real-world clinical data in the assessment of DAM thresholds and provide insight into how DAMs may be best applied in clinical practice. While it is evident that clinical improvement can occur at any level of disease activity, the benefit will be seen in patients with relatively higher disease activity, with the level of disease activity defined by our Youden-level assessment providing a good threshold level for consideration in clinical practice. Further work will be needed to determine if guidelines should be adjusted to include these findings in directing the treatment of RA.

## Conclusions

This work demonstrated that MTC was associated with clinical improvement across all DAMs, with the greatest change observed in patients with RA disease activity above the Youden threshold identified. Thus, the Youden level may be an important measure for consideration while making individualized treatment decisions.

## Supplementary information

**Additional file 1.** Supplementary tables and figures.

## Data Availability

The datasets supporting the conclusions of this article are available in the VA Corporate Data Warehouse.

## Abbreviations

ACR: American College of Rheumatology
ACR20 response: American College of Rheumatology criteria
CDW: Corporate Data Warehouse
CDAI: Clinical Disease Activity Index
CI: Confidence interval
DAMs: Disease activity measures
DAS28: Disease Activity Score for 28 joints
DMARD: Disease-modifying antirheumatic drug
EULAR: European League Against Rheumatism
GEE: Generalized estimating equation
MTC: Major therapeutic change
PAS II: Patient Activity Scale II
RD: Risk difference
RR: Risk ratio
RDCI: Rheumatic Disease Comorbidity Index
RF: Rheumatoid factor
RAPID3: Routine Assessment of Patient Index Data 3
SDAI: Simplified Disease Activity Index
SD: Standard deviation
VARA: Veterans Affairs Rheumatoid Arthritis

## References

[1] Singh JA, Saag KG, Bridges SL, Akl EA, Bannuru RR, Sullivan MC, Vaysbrot E, McNaughton C, Osani M, Shmerling RH (2016). 2015 American College of Rheumatology Guideline for the Treatment of Rheumatoid Arthritis. Arthritis Rheumatol.

[2] Smolen JS, Landewé R, Bijlsma J, Burmester G, Chatzidionysiou K, Dougados M, Nam J, Ramiro S, Voshaar M, van Vollenhoven R (2017). EULAR recommendations for the management of rheumatoid arthritis with synthetic and biological disease-modifying antirheumatic drugs: 2016 update. Ann Rheum Dis.

[3] Dougados M, Aletaha D, van Riel P (2007). Disease activity measures for rheumatoid arthritis. Clin Exp Rheumatol.

[4] Anderson J, Caplan L, Yazdany J, Robbins ML, Neogi T, Michaud K, Saag KG, O’Dell JR, Kazi S (2012). Rheumatoid arthritis disease activity measures: American College of Rheumatology recommendations for use in clinical practice. Arthritis Care Res (Hoboken).

[5] Stever JR, Cannon GW, Teng CC, Accortt NA, Collier DH, Sauer BC (2019). Utility of administrative and clinical data to predict major change in medical treatment in US veterans enrolled in the Veterans Affairs Rheumatoid Arthritis (VARA) registry. Clin Exp Rheumatol.

[6] Sauer BC, Chen W, Shen J, Accortt NA, Collier DH, Cannon GW. Major therapeutic changes have the potential to produce significant clinical response across a broad range of disease activity: an observational study of US veterans with rheumatoid arthritis. Arthritis Care Res (Hoboken). 2020. https://pubmed.ncbi.nlm.nih.gov/32166882/. 10.1002/acr.24183 32166882

[7] Youden WJ (1950). Index for rating diagnostic tests. Cancer.

[8] Mikuls TR, Fay BT, Michaud K, Sayles H, Thiele GM, Caplan L, Johnson D, Richards JS, Kerr GS, Cannon GW (2011). Associations of disease activity and treatments with mortality in men with rheumatoid arthritis: results from the VARA registry. Rheumatology (Oxford).

[9] Mikuls TR, Kazi S, Cipher D, Hooker R, Kerr GS, Richards JS, Cannon GW (2007). The association of race and ethnicity with disease expression in male US veterans with rheumatoid arthritis. J Rheumatol.

[10] Mikuls TR, Reimold A, Kerr GS, Cannon GW (2015). Insights and implications of the VA Rheumatoid Arthritis registry. Fed Pract.

[11] Fihn SD, Francis J, Clancy C, Nielson C, Nelson K, Rumsfeld J, Cullen T, Bates J, Graham GL (2014). Insights from advanced analytics at the Veterans Health Administration. Health Aff (Millwood).

[12] Felson DT, Anderson JJ, Boers M, Bombardier C, Furst D, Goldsmith C, Katz LM, Lightfoot R, Paulus H, Strand V (1995). American College of Rheumatology. Preliminary definition of improvement in rheumatoid arthritis. Arthritis Rheum.

[13] Fisher MC CD: Rheumatoid arthritis stable follow up visits - 3 month vs 6 month intervals. ACR/ARHP Annual Meeting; November 14-19; Boston, MA, 2014.

[14] Verhoeven AC, Boers M, van Der Linden S (2000). Responsiveness of the core set, response criteria, and utilities in early rheumatoid arthritis. Ann Rheum Dis.

[15] England BR, Sayles H, Mikuls TR, Johnson DS, Michaud K (2015). Validation of the rheumatic disease comorbidity index. Arthritis Care Res (Hoboken).

[16] Aletaha D, Smolen JS (2007). The Simplified Disease Activity Index (SDAI) and Clinical Disease Activity Index (CDAI) to monitor patients in standard clinical care. Best Pract Res Clin Rheumatol.

[17] Pincus T, Swearingen CJ, Bergman M, Yazici Y (2008). RAPID3 (Routine Assessment of Patient Index Data 3), a rheumatoid arthritis index without formal joint counts for routine care: proposed severity categories compared to Disease Activity Score and Clinical Disease Activity Index categories. J Rheumatol.

[18] Prevoo ML, van’t Hof MA, Kuper HH, van Leeuwen MA, van de Putte LB, van Riel PL: Modified disease activity scores that include twenty-eight-joint counts. Development and validation in a prospective longitudinal study of patients with rheumatoid arthritis. Arthritis Rheum 1995, 38(1):44–48. 10.1002/art.1780380107 7818570

[19] Snowden JM, Rose S, Mortimer KM (2011). Implementation of G-computation on a simulated data set: demonstration of a causal inference technique. Am J Epidemiol.

[20] Robins J (1986). A new approach to causal inference in mortality studies with a sustained exposure period—application to control of the healthy worker survivor effect. Mathematical Modelling.

[21] Liang K-Y, Zeger SL (1986). Longitudinal data analysis using generalized linear models. Biometrika.

[22] van Riel PL, Renskers L (2016). The Disease Activity Score (DAS) and the Disease Activity Score using 28 joint counts (DAS28) in the management of rheumatoid arthritis. Clin Exp Rheumatol.

[23] Tugwell P, Bombardier C (1982). A methodologic framework for developing and selecting endpoints in clinical trials. J Rheumatol.

[24] van Gestel AM, Prevoo ML, van’t Hof MA, van Rijswijk MH, van de Putte LB, van Riel PL: Development and validation of the European League Against Rheumatism response criteria for rheumatoid arthritis. Comparison with the preliminary American College of Rheumatology and the World Health Organization/International League Against Rheumatism criteria. Arthritis Rheum 1996, 39(1):34–40. 10.1002/art.1780390105 8546736

[25] Taylor PC, Alten R, Gomez-Reino JJ, Caporali R, Bertin P, Sullivan E, Wood R, Piercy J, Vasilescu R, Spurden D (2018). Clinical characteristics and patient-reported outcomes in patients with inadequately controlled rheumatoid arthritis despite ongoing treatment. RMD Open.

[26] Wei W, Sullivan E, Blackburn S, Chen CI, Piercy J, Curtis JR (2019). The prevalence and types of discordance between physician perception and objective data from standardized measures of rheumatoid arthritis disease activity in real-world clinical practice in the US. BMC Rheumatol.

[27] Reed GW, Collier DH, Koenig AS, Saunders KC, Pappas DA, Litman HJ, Kremer JM, Kotak S (2017). Clinical and demographic factors associated with change and maintenance of disease severity in a large registry of patients with rheumatoid arthritis. Arthritis Res Ther.

[28] Taylor PC G-RJ, Alten R, Bertin P, Caporali R, Sullivan E, et al.: Adoption of treat to target management in the context of achievable goals and satisfaction in RA [abstract]. Arthritis Rheumatol. 2015; 67 (suppl 10). https://acrabstracts.org/abstract/adoption-of-treat-to-target-management-in-the-context-of-achievable-goals-and-satisfaction-in-ra/. Accessed 23 July 2020.

