# Supplementary material for: Empirical evidence of disease activity thresholds used to indicate need for major therapeutic change in US veterans with rheumatoid arthritis
Source: Arthritis Res Ther. 2020 Oct 22;22:253. doi: 10.1186/s13075-020-02346-1 (PMC7579862; doi:10.1186/s13075-020-02346-1)
Supplement: Supplementary file 1 — Additional file 1. Supplementary tables and figures. [file 13075_2020_2346_MOESM1_ESM.docx]

**SUPPLEMENTAL MATERIALS**

**Empirical Evidence of Disease Activity Thresholds Used to Indicate Need for Major Therapeutic Change: An Observational Study in US Veterans with Rheumatoid Arthritis**

Grant W. Cannon, Wei Chen, Yizhe Xu, Jincheng Shen, Neil A. Accortt,
David H. Collier, Brian C. Sauer

**Supplemental Table S1:** Properties of disease activity measures

**Supplemental Table S2:** Type of MTC by disease activity measure and disease activity category

**Supplemental Figure S1:** Study design

**Supplemental Figure S2:** Youden thresholds for initiation of MTC by disease activity measure

**Supplemental Table S1.** Properties of disease activity measures

|  | **DAS28** | **CDAI** | **RAPID3** |
| --- | --- | --- | --- |
| Score range | 0–10 | 0–76 | 0–10 |
| Components | TJC SJC PtGA ESR | TJC SJC PtGA PGA | MDHAQ Patient pain score PtGA |
| Severity thresholds Remission Low Moderate High | < 2.6 ≥ 2.6 to < 3.2 ≥ 3.2 to ≤ 5.1 > 5.1 | ≤ 2.8 2.9 to 10.0 10.1 to 22.0 > 22.0 | ≤ 1.0 1.01 to 2.0 2.01 to 4.0 > 4.0 |

CDAI, Clinical Disease Activity Index; DAS28, Disease Activity Score with 28 joints; ESR, erythrocyte sedimentation rate; MDHAQ, Multidimensional Health Assessment Questionnaire; PGA, physician global assessment; PtGA, patient global assessment; SJC, swollen joint count; TJC, tender joint count; RAPID3, Routine Assessment of Patient Index Data 3.

**Table S2.** Type of MTC by DAM and disease activity category

|  | Study Population (12,094 Visits) | | | |
| --- | --- | --- | --- | --- |
| Disease activity measure category, n (%) | Change^a^ in oral prednisone | Joint injection | Change^a^ in bDMARD | Change^a^ in csDMARD^a^ |
| Overall | 832 (6.9) | 64 (0.5) | 641 (5.3) | 1,782 (14.7) |
| DAS28 Remission and low (< 3.20) Low moderate (3.20–4.02) High moderate (4.03–5.10) High (> 5.10) | 247 (4.7) 152 (6.2) 181 (7.6) 252 (12.7) | 10 (0.2) 10 (0.4) 23 (1.0) 21 (1.1) | 112 (2.1) 98 (4.0) 177 (7.4) 254 (12.8) | 506 (9.6) 316 (12.8) 474 (19.8) 486 (24.5) |
| CDAI Remission and low (< 10.0) Low moderate (10.0– 12.9) High moderate (13.0–22.0) High (> 22.0) | 255 (4.6) 78 (5.7) 215 (8.2) 284 (11.3) | 12 (0.2) 4 (0.3) 21 (0.8) 27 (1.1) | 116 (2.1) 36 (2.6) 160 (6.1) 329 (13.1) | 518 (9.3) 192 (14.0) 459 (17.5) 613 (24.5) |
| RAPID3 Remission and low (< 2.00) Low moderate (2.00–3.81) High moderate (3.82–4.00) High (> 4.00) | 116 (4.3) 177 (5.2) 33 (8.3) 506 (9.0) | 3 (0.1) 8 (0.2) 2 (0.5) 51 (0.9) | 66 (2.4) 113 (3.3) 19 (4.8) 443 (7.9) | 263 (9.8) 424 (12.4) 67 (16.9) 1,028 (18.4) |

^a^Changes included initiating medication, restarting medication after a gap, and/or change in medication dose.

bDMARD, biologic disease-modifying antirheumatic drug; csDMARD, conventional synthetic disease-modifying antirheumatic drug; CDAI, Clinical Disease Activity Index; DAS28, Disease Activity Score with 28 joints; MTC, major therapeutic change; RAPID3, Routine Assessment of Patient Index Data 3.

**Supplemental Figure S1.** Study schema.

**Supplemental Figure S2.** Youden-defined thresholds. The range of high disease activity (red), moderate disease activity (orange), low disease activity (blue), and remission (green) are shown for each disease activity measure. Dotted lines indicate the Youden-defined threshold that clinicians use in practice to initiate a major therapeutic change.
